# Role of Estrogen Receptors and G Protein-Coupled Estrogen Receptor in Regulation of Hypothalamus–Pituitary–Testis Axis and Spermatogenesis

**DOI:** 10.3389/fendo.2014.00001

**Published:** 2014-01-16

**Authors:** Adele Chimento, Rosa Sirianni, Ivan Casaburi, Vincenzo Pezzi

**Affiliations:** ^1^Laboratory of Applied Biology, Department of Pharmacy, Health and Nutrition Sciences, University of Calabria, Cosenza, Italy

**Keywords:** ESR1, ESR2, GPER, gonadotropins, HPG axis, spermatogenesis

## Abstract

Male reproductive function is under the control of both gonadotropins and androgens through a negative feedback loop that involves the hypothalamus, pituitary, and testis known as hypothalamus–pituitary–gonadal axis (HPG). Indeed, estrogens also play an important role in regulating HPG axis but the study on relative contribution to the inhibition of gonadotropins secretion exerted by the amount of estrogens produced within the hypothalamus and/or the pituitary or by the amount of circulating estrogens is still ongoing. Moreover, it is known that the maintenance of spermatogenesis is controlled by gonadotropins and testosterone, the effects of which are modulated by a complex network of locally produced factors, including estrogens. Physiological effects of estrogens are mediated by the classical nuclear estrogen receptor alpha and estrogen receptor beta, which mediate both genomic and rapid signaling events. In addition, estrogens induce rapid non-genomic responses through a membrane-associated G protein-coupled estrogen receptor (GPER). Ours and other studies reported that, in the testis, GPER is expressed in both normal germ cells and somatic cells and it is involved in mediating the estrogen action in spermatogenesis controlling proliferative and/or apoptotic events. Interestingly, GPER expression has been revealed also in the hypothalamus and pituitary. However, its role in mediating estrogen rapid actions in this context is under investigation. Recent studies indicate that GPER is involved in modulating gonadotropin-releasing hormone (GnRH) release as well as gonadotropins secretion. In this review, we will summarize the current knowledge concerning the role of estrogen/estrogen receptors molecular pathways in regulating GnRH, follicle-stimulating hormone, and luteinizing hormone release at the hypothalamic and pituitary levels in males as well as in controlling specific testicular functions such as spermatogenesis, focusing our attention mainly on estrogen signaling mediated by GPER.

## Introduction

Male fertility and hence its reproductive potential is a result of a complex and intricate as a fine neuroendocrine control. Traditionally the adult male reproductive function was considered to be controlled by both gonadotropins and androgens through a negative feedback loop that involves the hypothalamus, pituitary, and testis known as the hypothalamus–pituitary–gonadal axis (HPG). As such, spermatogenesis is regulated by the pulsatile release of gonadotropin-releasing hormone (GnRH) from the arcuate nucleus of the hypothalamus, which stimulates the anterior pituitary to release follicle-stimulating hormone (FSH) and luteinizing hormone (LH) (1). Accordingly, at the testicular level, LH stimulates the Leydig cells to produce testosterone, which has a local effect on the interstitium and seminiferous tubules and results in sperm production and maturation while FSH exerts its effect directly on the Sertoli cells that in turn promote and sustain spermatogenesis (1). Both GnRH and gonadotropin secretion could be modulated by testosterone and more surprisingly, estradiol (E2) acting on the hypothalamus or on the pituitary via a feedback regulating mechanisms (2). However, the specific role of each sex steroid in the regulation of gonadotropin negative feedback is still not completely clarified.

In males, the major source of circulating estrogens is the aromatization of androgens as a consequence of the action of the enzyme complex known as aromatase that is widely expressed in a number of male tissues including the testis and brain (3, 4).

Cellular effects of estrogens occur via classical estrogen receptor alpha (ESR1) and estrogen receptor beta (ESR2) located in the nucleus and cytoplasm of the target cells and belong to the nuclear receptor superfamily members that act as nuclear transcription factors, binding to estrogen response elements (EREs) within specific genes to alter their rate of transcription (5). However, it has become clear that estrogens also exert rapid, non-genomic effects by altering different signaling pathways both in central and nervous system peripheral tissues (6).

These “non-genomic effects” could be mediated by extranuclear estrogen receptors (ERs) or by non-classical membrane bound receptors such as G protein-coupled estrogen receptor also named GPR30/GPER that has been identified as a novel ER (7). Estradiol through GPER rapidly activates different pathways including the stimulation of adenylyl cyclase, mobilization of intracellular calcium (Ca^2+^) stores, and activation of mitogen-activated protein kinase (MAPK) and phosphoinositide 3-kinase (PI3K) signaling pathways (8, 9).

In this review, we will summarize the current knowledge concerning the role of estrogen/ERs signaling in regulating GnRH, FSH, and LH release at the hypothalamic and pituitary levels in males as well as in controlling specific testicular functions such as spermatogenesis, focusing our attention mainly on estrogen signaling mediated by GPER.

## Role of Estrogen and Estrogens Receptors in GnRH, LH, and FSH Secretion in Males

### Estrogen functions at the hypothalamic level

Gonadotropins and gonadal steroids, being involved in the regulation of secondary sex characteristics, gametogenesis, cellular functions, and also behavior, are the main driving force for reproductive function. The hypothalamic GnRH neurons that control LH and FSH release from the pituitary represent the final common pathway for neuronally derived endogenous as well as exogenous stimuli (10). In both males and females, gonadal steroid hormones exert negative feedback regulation on HPG axis activity at both the hypothalamus and pituitary levels. In females, the feedback mechanism is more complex since estrogen and progesterone induce both negative and positive feedback responsible for generating the pre-ovulatory GnRH and LH surge (10). Thus, the neuroendocrine mechanism underlying the ovulatory LH and FSH surge, characteristic of the mature female reproductive system, is usually extinguished in males by neonatal androgen imprinting (10).

Several evidences indicate that testicular steroids, androgens, and estrogens could mediate the feedback actions on gonadotropin secretion interacting with their receptors, ERs or androgen receptors (ARs) that were found in the male hypothalamus (11). However, there is no clear consensus on the role of ER versus AR signaling in males (12, 13). Aromatization of testosterone to estradiol and reduction to 5α-dihydrotestosterone (DHT) is mandatory for normal male reproduction and occurs in peripheral (14) and central tissues (15, 16). Sharma and co-workers have demonstrated that aromatase inhibitor administration into the third cerebral ventricle of intact rams resulted in an increased frequency of LH pulses without affecting estradiol plasma concentrations (17). In addition, existence of these feedback actions is further clearly illustrated in a range of species by an increased secretion of the gonadotropins following castration (18–20). Accordingly, an increased LH secretion was found also in intact or castrated rams passively or actively immunized against estradiol (18). However, how testosterone and/or its primary metabolites act within the brain to suppress the synthesis and/or secretion of GnRH need more investigation.

In humans, androgen aromatization for normal gonadotropins feedback function (21) has been discovered by the use of testosterone or estradiol infusion in men affected by idiopathic hypothalamic hypogonadism (IHH). On the other hand, the authors did not record any change in LH and FSH secretion when pure androgen DHT was administered. These data indirectly suggest that the peripheral 5α-reduction of testosterone to DHT plays a minor role in the control of the secretion of gonadotropins (21). Thus, the inhibitory effect on gonadotropin secretion is mediated mainly by estradiol from endogenous conversion of testosterone rather than direct androgen action, at least in the pituitary gland (21). Indeed, other studies suggested that *in situ* aromatization of testosterone is required both at the hypothalamic and pituitary levels to insure a complete feedback mechanism of gonadotropins (22, 23). Moreover, the results coming from basal, GnRH-stimulated, and pulsatile evaluation of LH and FSH secretion in two aromatase-deficient men have provided direct evidence that circulating estrogens exert an inhibitory control in LH feedback at both the hypothalamic and pituitary levels (24).

It is universally accepted that estradiol actions were mediated by its interaction with ERs ESR1 and ESR2 that act as hormone-inducible transcription factors determining estrogen-dependent gene transactivation (1). Several studies, involving a range of species and both sexes, have demonstrated that GnRH neurons do not express ESR1 (25–27), even though a small number of GnRH neurons containing ESR1 were found in female rats (28). Indeed, accumulating evidence suggests that estrogen could act in GnRH neurons through ESR2. In fact, ESR2 immunoreactivity was detected first in rodents (29, 30) and later in humans (31). However, studies performed in *Esr1* knock-out mice suggest that in males, ESR1 is the predominant receptor involved in mediating estradiol suppression of GnRH content (12). Moreover, it was also demonstrated that in mouse LHRH neurons (29) ESR2 may mediate the rapid estradiol effects because mouse LHRH neurons expressed only ESR2, and the nuclear ER antagonist, ICI 182,780, suppressed the effect of estradiol on Ca^2+^ oscillations. However, in primate LHRH neurons, estradiol appears to cause its action through a different mechanism, because ICI 182,780 failed to block the estradiol-induced changes in Ca^2+^ oscillations and synchronization (32). This finding could be explained by the study of Noel and co-workers (33) suggesting a GPER involvement in the rapid action of estradiol in hypothalamic neurons. In fact these authors demonstrated that GPER is expressed in olfactory placode cultured cells and in a subset of LHRH neurons and that GPER gene knockdown in LHRH neurons completely abrogate both estradiol- and estrogen-dendrimer conjugate-induced changes in Ca^2+^ oscillations. Furthermore, using a selective specific GPER-agonist, they obtained changes in Ca^2+^ oscillations similar to those observed upon estradiol treatment confirming that estradiol rapid action appears to be mediated, at least partially, through GPER (33). However, further investigation is needed to better clarify what the specific target cells for estrogens action at the hypothalamic level are and what receptors are involved.

### Estrogen functions at the pituitary level

In male vertebrates, LH and FSH plasma levels are largely regulated by GnRH and activins as stimulators and steroids and inhibins as inhibitors (34, 35). The negative feedback action of testicular androgens on serum LH and FSH was first demonstrated utilizing castrated animal models evidencing a substantial increase in LH and FSH levels that were prevented by the administration of physiological levels of testosterone (36). Later studies have pointed out the hypothalamus and pituitary as targets for such feedback. Although there are conflicting data concerning the effects of testosterone on GnRH synthesis and secretion, studies have demonstrated that castration and steroid replacement alter levels of GnRH messenger RNA (mRNA) (37), processing of GnRH prohormone (38), hypothalamic GnRH contents (39), and patterns of pulsatile GnRH release (39, 40). Besides examining hypothalamic sites of action, a number of investigators have also examined feedback directly on the pituitary. Testosterone, DHT, or estradiol is able to suppress GnRH-stimulated LH secretion from pituitary cultures (41), whereas T treatments increase basal FSH secretion and intrapituitary FSH levels (42). Furthermore, molecular analyses of the promoter regions of the gonadotropin genes such as α-gonadotropin subunit (αGSU), FSHβ, and LHβ subunits (43) have revealed the presence of responsive elements through which AR or ER mediated the feedback effects exerted by testosterone or estradiol, respectively.

It is worth noting that estrogen responsiveness of the pituitary gland requires the presence of ERs, including the classical ESR1 and ESR2 (44). The ER expression and distribution patterns in pituitary glands have been studied in rats (45), sheep (46), and humans (47). The localization of ARs in the pituitary is also well-established since AR expression has been reported in the anterior pituitary gland of humans (48), rhesus monkeys, rats (49), Brazilian opossums (50), and mice (51).

Although these data support pituitary sites of steroid action, mainly in feedback regulation, it is unclear whether the effects of T are primarily mediated directly through the AR or indirectly via aromatization and activation of ERs. Experiments performed with a non-aromatizable androgen DHT has been demonstrated to suppress serum LH and basal levels of αGSU and LHβ mRNA in rats (52), confirming AR-mediated feedback. As such, antiandrogen flutamide induced up-regulates of LH serum concentrations (53). At the molecular levels it was also demonstrated that the enhancer elements of the αGSU gene is a target of AR-mediated suppression (43).

In addition, other studies have demonstrated that exogenous estradiol treatment (34) reduced LH and FSH concentrations and gonadotropin mRNAs content, while treatment with aromatase inhibitors determines an increase of LH serum levels (54). The roles of estrogens/ESR1 signaling are further supported clinically by the elevated serum FSH levels in an estrogen-resistant patient (55) as well as in aromatase-deficient humans (24). The unsolved debate focusing on what steroid receptor, AR and/or ESR1, is able to mediate negative feedback on serum gonadotropins is further complicated by the presence of ESR2 (56). Although ESR2 mRNA levels are very low in adult mouse pituitaries (57), there are studies, as already above mentioned, reporting that the hypothalamic nuclei of both rats and mice express ESR2 at both transcriptional and post-transcriptional levels (57, 58). Thus, it is reasonable to hypothesize that testicular steroids could modulate hypothalamic-pituitary activity directly through AR or indirectly through aromatization and activation of either ESR1 or ESR2 signaling pathways.

Estradiol effects in the pituitary gland occur mainly through genomic mechanisms (59) as evidenced in a mouse gonadotroph cell line (LβT2) where estradiol administration increased LHβ mRNA levels (60) due to the presence of EREs within the promoter region of LHβ gene (61). It is noteworthy that there is also experimental evidence for estrogen-independent ESR1 transcriptional activation in gonadotrope cells most probably through GnRH receptor and signaling via protein kinase C (PKC) and MAPK pathways (62). Recent studies indicate that GPER is involved in suppressing GnRH-stimulated LH release in primary pituitary cell culture derived from ovariectomized ewes (63). However, to date there are no studies showing GPER-mediated non-genomic signaling events in the male pituitary. Since GPER has been identified in the plasma membrane of a variety of target tissues, including anterior pituitary (64, 65), we can speculate that GPER could have a role in mediating the non-genomic effects of estradiol in the male pituitary.

## Estrogen and HPG Axis in Males: Lessons from Animal Models

The development of knock-out or transgenic mice with targeted disruptions of ERs and/or aromatase has increased our understanding of estrogen function in reproduction (66).

Controversy aspect regarding the male hypothalamic and pituitary feedback regulation by steroids has been partially resolved by the observation of data coming from the castration and steroids replacement experiments in *Esr1* knock-out (ERKO) mouse (67) model. Lindzey and co-workers demonstrated that in males, ESR1 is the predominant receptor involved in mediating estradiol suppression of gonadotropin release and gonadotropin subunit mRNA expression (12). The role of an activated AR by testosterone is, of course, not secondary, as demonstrated by the ability of testosterone administration to suppress serum LH in ERKO male mice but its aromatization seems to produce a more functional inhibitory effect on the hypothalamic-pituitary feedback and this is also true for FSH production (12).

Other *in vivo* studies confirmed that estrogens have important roles in the regulation of spermatogenesis. The hypogonadal (*hpg*) mouse (68) that does not produce mature GnRH decapeptide due to a truncation in the GnRH gene is widely used as an animal model to investigate the endocrine regulation of spermatogenesis (69). *Hpg* mice are infertile because they do not produce gonadotropins and hence the testis failed to develop (70). By the *hpg* mice model it was demonstrated that treatment with LH stimulate steroidogenesis (71) and a combined treatment with FSH and androgens induce normal spermatogenesis (72, 73). More interestingly, later research demonstrated that chronic estradiol treatment of this animal model was able to restore spermatogenesis (69, 74, 75), via a mechanism involving a weak neuroendocrine activation of FSH secretion. These latter results raised the question about the site specific action of estrogen in *hpg* mouse model. Further studies based on traditional pharmacological approaches using selective ER agonists in engineered *hpg* animals knocked-out for ERs (*hpg*/ESR1 and *hpg*/ESR2) revealed that estradiol-mediated spermatogenesis takes place in *hpg* animals through the involvement of ESR1, but not ESR2, dependent mechanism responsible for the increase of FSH and testis (mainly Sertoli cells) function.

Spermatogenesis as a target for estrogen/ER signaling has been documented by the use of knock-out mice model for all three ERs (ESR1, ESR2, and GPER) as well as for the aromatase gene. *Esr1* KO animals have reduced fertility because of abnormal fluid reabsorption in the efferent ductules (76), whereas initially spermatogenesis, steroidogenesis, and fertility were found unaffected in *Esr2* KO animals (66). However, all these *Esr2* mutants displayed alternative splicing transcripts that could compensate for the lack of full-length receptor isoform. An interesting study showed that a new *Esr2*^−/−^ mutant mouse, in which exon 3 of *Esr2* was deleted by Cre/LoxP-mediated excision, completely avoiding any downstream transcripts, produced sterile males (77). The cause for the sterility of these male mice is still unknown, because their gonads and internal genital organs appear *histologically* normal and the mobility of their spermatozoa appears normal too (77). In aromatase knock-out (ArKO) mice the lack of estrogen production results in an alteration of a complex hormonal balance controlling meiosis progression, leading to a significant decrease in spermatocytes and round and elongated spermatids number associated with apoptotic features (78, 79). The more severe testicular phenotype observed in ArKO mice compared to ERKO mice (66) supports the hypothesis that an alternative receptor (i.e., GPER) and alternative pathways could be involved in mediating the effects of estrogen on spermatogenesis.

A study with *Gper* deficient mice (80) claimed that Gper was not involved in estrogenic responses of reproductive organs. However, even though male and female *Gper* KO mice were found fertile, it is noteworthy that the study did not show data on the spermatogenetic process, while a careful examination of estrogenic response was carried out only on the uterus and mammary glands.

A mouse model harboring a two amino-acid mutation of the DNA-binding domain (E207A, G208A) that precludes direct binding of ESR1 to an ERE has allowed discrimination between estrogen action through ERE versus non-ERE pathways (81). The loss of non-classical ESR1 signaling pathways is responsible for most of the reproductive tract defects observed in male ERKO mice (81). These data do not, however, distinguish between the various non-classical pathways (e.g., tethering versus membrane signaling) but support strongly the hypothesis that rapid estrogen signaling could play a crucial role in spermatogenesis.

An original study using estrogen non-responsive *Esr1* knock-in (ENERK1) mice, which have a point mutation in the LBD of *Esr1* that significantly reduces interaction with and response to endogenous estrogens, but does not affect activation of Esr1 by growth factors, showed that estrogen-dependent Esr1 signaling is required for germ cell viability (82).

New information on the role of ESR1 signaling in the regulation of chromatin remodeling during spermiogenesis were obtained from recent works on Type 1 Cannabinoid Receptor Knock-out Mice (*Cnr1*^−/−^) model by Cacciola et al. (83, 84). The characterization of the reproductive *Cnr1*^−/−^ Mice phenotype [reviewed in Ref. (85)] revealed that estrogen through its receptor is able to preserve chromatin condensation and DNA integrity of spermatozoa by promoting histone displacement in spermatids.

In summary, the studies *in vivo* support the findings that estrogen and its major receptor, ESR1, have important roles in the regulation of spermatogenesis, particularly with aging (86) and that this activity occurs through both rapid non-classical membrane-associated/growth factor receptors as well as classical transcriptional mediated pathways. Future studies are required to better understand the separation of these pathways and their potential interactions with other steroid receptors that coexist in the same cell types.

## Estrogen and Estrogen Receptors in Spermatogenesis

Spermatogenesis, which takes place in the seminiferous epithelium, can be divided into three major steps: spermatogonia proliferation by mitosis, formation of preleptotene spermatocytes which then gives birth to round spermatids (RSs) via meiosis, and spermiogenesis that allows the maturation of spermatids into mature spermatozoa. This complex and coordinated process is regulated by numerous endocrine, paracrine, or autocrine factors (87, 88) including gonadotropins LH and FSH, androgens, and estrogens (86, 89, 90).

It is known that estrogen action mediated by its specific receptors, such as ESR1, ESR2, and GPER, has different localization and expression through the entire mammalian male reproductive tract (86, 91) with major differences between species, as well as between individuals belonging to the same species (86). In mouse testis, ESR1 was found in Leydig cells, in some peritubular myoid cells (92, 93), and in Sertoli cells (94), whereas ESR2 was found in Leydig cells, Sertoli cells, and some germ cells, particularly spermatocytes (92, 93). In the rat, ESR1 immunodetection was restricted to the Leydig cells (95), in immature rat Sertoli cells (94, 96), in the seminiferous compartment (97), and in purified germ cells (98, 99). Regarding ESR2, there is a general consensus concerning its localization in seminiferous tubules but conflicting data regarding its presence in germ cells (86, 100) although Bois and co-workers detected the presence of ESR2 in pachytene spermatocytes (PS) and RSs (101). The presence of ERs in testicular cells of humans is well documented (90, 102). The two types of ERs, 1 and 2, have been identified in isolated immature germ cells in men, the full-length protein ESR1 (66 kDa) and one isoform lacking the exon 1 (46 kDa). In mature spermatozoa, only the 46-kDa band was observed. For ESR2, two proteins that correspond to the long (60 kDa) and short (50 kDa) forms have been detected in germ cells (102). However, the presence of ESR1 and ESR2 in the human ejaculated spermatozoa has been demonstrated (90, 103).

Recently, ours and other studies have demonstrated the presence of a functional GPER in both normal (98, 99, 104, 105) and malignant testicular cell lines (106).

The important role of estrogens in spermatogonial cell proliferation has been evidenced by works of Chieffi et al. where the authors demonstrated at the molecular level the involvement of ERK/c-fos signaling (107, 108). Accordingly, studies with the mouse spermatogonial GC-1 cell line showed that estradiol rapidly activates EGFR/ERK/fos/cyclin D1 pathway through a functional cross-talk between GPER and ESR1 responsible for cell proliferation (104). Conversely, estradiol-mediated rapid ESR1 and/or GPER/EGFR/ERK/c-jun pathway activation in primary cultures of rat PS (98) and in GC-2 cells (105), an immortalized mouse pachytene spermatocyte-derived cell line, induces an apoptotic mechanism. In particular, in PS cells GPER activation is related to a reduction of cyclin A1 and B1 expression concomitantly with an increase of bax protein expression (98), while in GC-2 cells GPER signaling is associated with the phosphorylation of all MAPK family members initiating the intrinsic apoptotic pathway (105). Similarly, a functional cross-talk between ESR1 and GPER in mediating apoptotic effects was observed also in primary cultures of adult rat RSs (99). It is noteworthy that in this cellular context, the contribution of ESR2 seems to be related to anti-apoptotic events (99).

G protein-coupled estrogen receptor expression and signaling was also investigated in cultured immature rat Sertoli cells (109, 110) where it has been observed that ERs are able to regulate gene expression involved in both cell proliferation and apoptosis. Indeed, ESR1 activated by its ligand rapidly induces EGFR/ERK1/2 and PI3K pathways that in turn increase cyclin D1 expression responsible for Sertoli cell proliferation (111). Interestingly, through the same molecular pathways the activation of GPER determines anti-apoptotic events by upregulating BCL2 and BCL2L2 proteins. Alternatively, the anti-apoptotic effects could be mediated by estradiol or G-1-GPER/EGFR/ERK1/2/pCREB dependent pathway driving a decrease of bax expression (111).

All these data evidenced that ERs and GPER through different molecular signaling may mediate estradiol action important for the function and maintenance of testicular cells where the complex balance between cellular maturation and cell death drive spermatogenesis and male (in)fertility.

Regarding GPER role in malignant testicular cell lines it has been shown that it is highly expressed in testicular germ cell cancer (TGCC) (112) as well as in Leydig and Sertoli cell tumors (113–115). However, also in this context, GPER activity appears to be cell type specific. In fact, in human testicular seminoma cell line, GPER activation is associated with increased cell proliferation (116), while in rat tumor, Leydig cell line is related to cell growth inhibition and apoptosis (106).

## Concluding Remarks

The reproductive hormonal axis in males normally functions in a tightly regulated manner to produce concentrations of circulating steroids required for normal male sexual development, sexual function, and fertility. The testis has the ability to also produce significant amounts of estrogenic hormones and a regulated balance between androgens and estrogens seems to be essential for normal testicular physiology and reproduction acting both within the testis as well as in regulating HPG axis.

Studies discussed in this review have suggested that estradiol is the main hormone that provides negative feedback at the hypothalamic level, whereas the pituitary requires both estradiol and DHT for a complete negative feedback effect. However, further investigation is necessary to better understand how testosterone and/or its primary metabolites act within the brain to suppress the synthesis and/or secretion of GnRH. Accumulating evidence suggests that estrogen could act in the hypothalamus through rapid action mediated by ESR2, and at least partially, through GPER (33). However, it remains to establish: (i) the specific target cells (GnRH neurons, glia cells, etc.) for estrogen action at the hypothalamic level; (ii) the ER isoforms involved; (iii) the signal transduction activated by estrogen in the different cell types. An unsolved debate is focused on clarifying what steroid (DHT and/or E2) and consequently what steroid receptors (AR and/or ESR1, ESR2) are able to induce and mediate negative feedback at the pituitary level. Interesting studies using engineered *hpg* animals knocked-out for ERs (*hpg*/ESR1 and *hpg*/ESR2), revealed that estradiol-mediated spermatogenesis takes place in *hpg* animals through the involvement of ESR1, but not ESR2, which increases FSH release and testis (mainly Sertoli cells) functions. However, the debate on negative feedback at the pituitary level is further complicated by recent observations that GPER could be involved in suppressing GnRH-stimulated LH release in primary pituitary cell culture derived from ovariectomized ewes (63). However, to date, there are no studies showing GPER-mediated non-genomic signaling events in the male pituitary.

Another important finding is that estrogen plays a direct role in modulating spermatogenesis influencing, in a cell specific manner, germ cells proliferation, differentiation, as well as germ cell survival and apoptosis. The widespread presence of ESR1 and ESR2 in all testicular cells supports this finding and the discovery of GPER in the testis has opened new perspectives to better understand the rapid membrane pathways induced by estrogens. In fact, estrogenic activity in the testis as well as at the hypothalamic level appears to involve not only the classical genomic pathway, but also rapid membrane receptor initiated pathways. Studies discussed in this review indicate the ability of ERs to trigger rapid and converging pathways controlling proliferation (i.e., proliferation through ESR1 and GPER in spermatogonia or apoptosis through the same receptors in spermatids); or trigger, independently from each other, pathways controlling the same cell function (i.e., apoptosis through ESR1 and/or GPER in spermatocytes). Moreover, these studies support the hypothesis that in the testis, as in other tissues, estrogen effects are a result of the combination of different ER mediated activities, including the classic genomic as well as rapid actions at the membrane receptors via a functional cross-talk with growth factor receptors.

Another interesting aspect is that genomic and rapid pathways can work independently from each other but at same time cooperate to reach a common goal (i.e., in Sertoli cells E2-genomic action on cyclin D1 induces proliferation and estradiol rapid action through GPER activates anti-apoptotic signals).

Further studies are necessary to clarify the role of estrogen/ERs signaling in regulating GnRH, FSH, and LH release at the male hypothalamic and pituitary levels as well as in controlling spermatogenesis. Such studies could be helpful to better understand the impact of environmental endocrine disruptors’ exposure, such as xenoestrogens, on male reproduction. In addition, more investigation is required to clarify the molecular mechanisms related to estrogen-dependent testicular tumorigenesis as well as to also provide a potential target for the development of a non-androgen male contraceptive.

## Conflict of Interest Statement

The authors declare that the research was conducted in the absence of any commercial or financial relationships that could be construed as a potential conflict of interest.
